# Restricted- and over-feeding during gestation decreases growth of offspring throughout maturity

**DOI:** 10.1093/tas/txad061

**Published:** 2023-05-31

**Authors:** Nicole M Tillquist, Sarah A Reed, Mia Y Kawaida, Amanda S Reiter, Brandon I Smith, Hyung Jang, Ji-Young Lee, Elaine C Lee, Steven A Zinn, Kristen E Govoni

**Affiliations:** Department of Animal Science, University of Connecticut, Storrs, CT 06269, USA; Department of Animal Science, University of Connecticut, Storrs, CT 06269, USA; Department of Animal Science, University of Connecticut, Storrs, CT 06269, USA; Department of Animal Science, University of Connecticut, Storrs, CT 06269, USA; Department of Animal Science, University of Connecticut, Storrs, CT 06269, USA; Department of Nutritional Sciences, University of Connecticut, Storrs, CT 06269, USA; Department of Nutritional Sciences, University of Connecticut, Storrs, CT 06269, USA; Department of Kinesiology, University of Connecticut, Storrs, CT 06269, USA; Department of Animal Science, University of Connecticut, Storrs, CT 06269, USA; Department of Animal Science, University of Connecticut, Storrs, CT 06269, USA

**Keywords:** growth, metabolism, maternal nutrition, residual feed intake, sheep

## Abstract

To determine the effects of poor maternal nutrition on the growth and metabolism of offspring into maturity, multiparous Dorset ewes pregnant with twins (*n* = 46) were fed to either 100% (control; *n* = 13), 60% (restricted; *n* = 17), or 140% (over; *n* = 16) of National Research Council requirements from day 30 ± 0.02 of gestation until parturition. Offspring of these ewes are referred to as CON (*n* = 10 ewes; 12 rams), RES (*n* = 13 ewes; 21 rams), or OVER (*n* = 16 ewes; 13 rams), respectively. Lamb body weights (BW) and blood samples were collected weekly from birth (day 0) to day 28 and then every 14 d until day 252. Intravenous glucose tolerance test (infusion of 0.25 g dextrose/kg BW) was performed at day 133 ± 0.25. At day 167 ± 1.42, individual daily intake was recorded over a 77 d feeding period to determine residual feed intake (RFI). Rams were euthanized at day 282 ± 1.82 and body morphometrics, loin eye area (LEA), back fat thickness, and organ weights were collected. The right leg was collected from rams at necropsy and dual-energy x-ray absorptiometry was used to determine bone mineral density (BMD) and length. Averaged from day 0 until day 252, RES and OVER offspring weighed 10.8% and 6.8% less than CON offspring, respectively (*P* ≤ 0.02). When adjusted for BW, liver and testes weights tended to be increased and decreased, respectively, in RES rams compared with CON rams (*P* ≤ 0.08). Additionally, RES BMD and bone length were less than CON rams (*P* ≤ 0.06). Treatment did not influence muscle mass, LEA, or adipose deposition (*P ≥* 0.41). Rams (−0.17) were more feed efficient than ewes (0.23; *P <* 0.01); however, no effect of maternal diet was observed (*P ≥* 0.57). At 2 min post glucose infusion, glucose concentrations in OVER offspring were greater than CON and RES offspring (*P* = 0.04). Concentrations of insulin in CON rams tended to be greater than OVER and RES ewes at 5 min (*P* ≤ 0.07). No differences were detected in insulin:glucose or area under the curve (AUC) for glucose or insulin (*P* ≤ 0.29). Maternal diet did not impact offspring triglycerides or cholesterol (*P* ≤ 0.35). Pre-weaning leptin tended to be 70% greater in OVER offspring than CON (*P* ≤ 0.07). These data indicate that poor maternal nutrition impairs offspring growth throughout maturity but does not affect RFI. Changes in metabolic factors and glucose tolerance are minimal, highlighting the need to investigate other mechanisms that may contribute to negative impacts of poor maternal diet.

## Introduction

The livestock industry is a multi-billion-dollar industry that supplies essential commodities such as meat, dairy products, eggs, and wool. United States beef and sheep production are projected to decrease in 2023 due to drought and increased cost of production (Knight et al., 2022). Efficiency of livestock production is critical to provide high-quality protein sources to sustain the growing world population and maintain industry profitability. Poor maternal nutrition during gestation can result in offspring that have decreased muscle mass (Reed et al., 2014; Du et al., 2017), increased adiposity (Ford et al., 2007; Long et al., 2015), decreased gain to feed ratio (G:F; Neville et al., 2010) and average daily gain (ADG; Hoffman et al., 2014, 2016; Van Emon et al., 2015), and impaired metabolism (Hoffman et al., 2014; Smith et al., 2021) demonstrating that nutritional insult of the dam can generate offspring that are less efficient and produce decreased quality product, ultimately impacting the efficiency of livestock production to provide high quality protein.

The effects of inadequate nutrition during gestation on offspring growth and development begin in utero and contribute to metabolic dysregulation of offspring into maturity (Barker, 1997). It is well established that restricted- and over-feeding during gestation can alter offspring body weight (BW; Hoffman et al., 2014, 2016; Liu et al., 2015; Pillai et al., 2017). However, changes in body composition as a result of poor maternal diet can be observed independent of BW and result in decreased muscle mass and increased adiposity which may negatively impact the health and productivity of the offspring (Ford et al., 2007; Long et al., 2011; Shasa et al., 2015). Understanding mechanisms that contribute to this will allow for methods to improve animal production (Reed and Govoni, 2017). However, the mechanisms that result in altered offspring phenotypes are not well characterized in offspring at maturity. We hypothesized that restricted- and over-feeding during gestation will reduce offspring BW, feed efficiency, muscle mass, glucose tolerance, alter circulating metabolic factors, and increase offspring adipose deposition. Therefore, the objective of this study was to expand on previous findings from our lab and others to evaluate the effects of poor maternal nutrition (restricted- and over-feeding during gestation) on offspring growth and metabolism from birth into maturity.

## Materials and Methods

### Animal Management

All procedures were completed in accordance with guidelines established and approved by the University of Connecticut Animal Care and Use Committee (A22-017).

**Figure 1.**
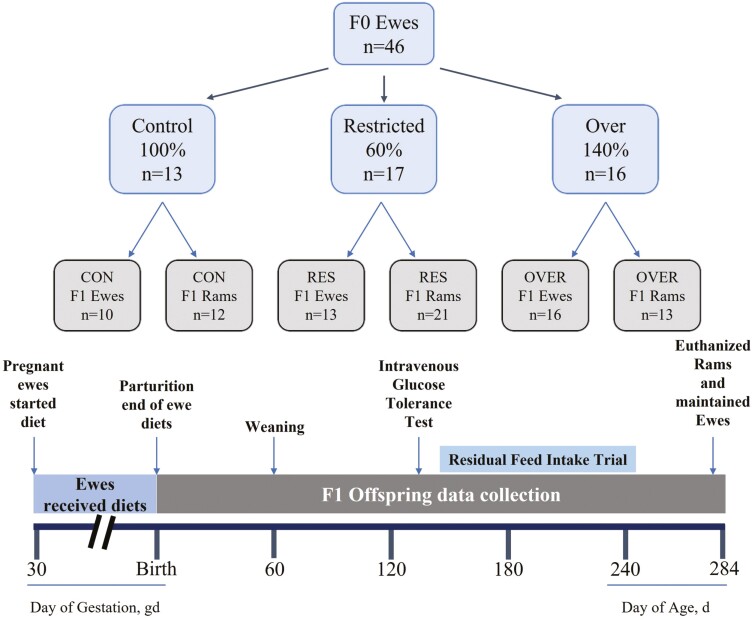
Experimental design. To evaluate the effects of restricted- and over-feeding on postnatal offspring development, pregnant ewes (*n* = 46) were housed in individual pens on day 20 of gestation and transitioned to a control complete pelleted diet. At day 26 of gestation ewes were blocked by BW and assigned to one of three dietary treatments (Control: 100%; Restricted: 60%; or Over: 140% of NRC requirement for TDN for ewes pregnant with twins). Offspring were weaned at day 60 and underwent intravenous glucose tolerance tests and residual feed intake trials at day 133 and 168 of age, respectively. Ram offspring were necropsied at day 282 of age, and ewes were maintained on a control diet for future breeding.

Multiparous Dorset ewes (F0; *n* = 46; Figure 1) were estrus synchronized using prostaglandin (Lutalyse, Pfizer Animal Health, New York, NY) and controlled intravaginal drug release devices (CIDRs; Zoetis, Parsippany-Troy Hills, NJ) and bred by live cover with one of three genetically related Dorset rams as previously described (Reed et al., 2014; Hoffman et al., 2016; Pillai et al., 2017). Day 0 of pregnancy was considered when a raddle mark was observed on the rump of the ewe. If the ewe was not remarked by day 20 of gestation, she was moved to an individual pen and transitioned to a complete pelleted feed (Table 1). Pregnancy with twins was confirmed using transabdominal ultrasound (Jones et al., 2016). Two F0 ewes were pregnant with triplets, where the third offspring (balanced by BW) was removed from the dam at birth so only twins were nursing. Three ewes were removed from the study for reasons unrelated to treatment (Control, *n* = 2; Over, *n* = 1; not included in figures. or statistical analysis). Ewes were fed a complete pelleted feed (Table 1) to meet 100% (control-fed), 60% (restricted-fed), or 140% (over-fed) of total digestible nutrients based on National Research Council (NRC) requirements for ewes pregnant with twins from day 30 of gestation through parturition. Diets began at day 30 of gestation to be consistent with previous studies and allow for confirmation of fetus number. Body weights and body condition scores (BCS; Russel, 1984) were measured weekly. Feed was adjusted based on individual BW on a weekly basis. Ewes were allowed to lamb, and after lambing were fed 100% of NRC requirements for lactating ewes so that the nutritional insult can be attributed to maternal gestational diet only. Resulting offspring are referred to as control (CON), restricted (RES), or over (OVER) corresponding to the diets of their dam. Nine offspring did not complete the study due to reasons unrelated to the experiment (CON, *n* = 3; RES *n* = 4; OVER, *n* = 2; not included in figures or statistical analysis). After birth, lambs were maintained with their dam and allowed ad libitum access to creep feed (Table 1; Home Fresh 18 Sheep Starter, Blue Seal, Litchfield, CT) and second cutting hay until weaning at 60 d of age. After weaning (Figure 1), lambs were housed together and fed grower feed (Table 1; Home Fresh Shepherd 16, Blue Seal) 100% of NRC requirements. An intravenous glucose tolerance test (IV-GTT) was performed on lambs at 133 ± 0.25 d of age, as previously described (Ford et al., 2007; Hoffman et al., 2014). To evaluate offspring feed efficiency during a rapid period of growth, residual feed intake (RFI) was measured in ram and ewe lambs using a Super SmartFeed system (C-Lock Inc., Rapid City, SD) and complete pelleted feed (Table 1) at day 167 ± 1.42 of age. To evaluate offspring composition at maturity, ram lambs were euthanized at day 282 ± 1.82 of age, and ewe lambs were maintained on a control diet.

**Table 1.** Chemical composition of F0 ewe and F1 offspring diets

	F0 Ewes	Offspring		
Nutrient analysis^1^	Complete pellet^2^	Creep feed^3^	Grower feed^4^	Complete pellet^5^
Moisture, %	11.96	10.80	13.50	9.45
Dry matter, %	88.01	89.20	86.60	90.57
Crude protein, %	13.38	21.20	18.50	18.48
Adjusted crude protein, %	13.38	21.20	18.50	18.48
ADF, %	28.19	11.70	11.70	25.95
aNDF, %	38.52	18.80	24.70	40.28
TDN, %	74.75	81.00	79.00	74.33
DE, Mcal/kg	2.87	3.37	3.26	2.80
NE_L_, Mcal/kg	1.76	1.91	1.87	1.75
NE_M_, Mcal/kg	1.78	2.00	1.94	1.77
NE_G_, Mcal/kg	1.16	1.34	1.30	1.15
Calcium, %	1.34	0.75	1.45	1.42
Phosphorus, %	0.42	0.63	0.68	0.54
Magnesium, %	0.25	0.28	0.32	0.34
Potassium, %	1.19	1.58	1.00	2.16
Sodium, %	0.18	0.25	0.35	0.51
Iron, mg/kg	641.75	394.00	270.00	358.50
Zinc, mg/kg	128.33	316.00	187.00	103.17
Copper, mg/kg	13.50	6.00	4.00	11.67
Manganese, mg/kg	82.25	158.00	125.00	86.33
Molybdenum, mg/kg	3.99	12.50	0.50	5.83
Sulfur, %	0.32	0.45	0.42	0.29

^1^Nutrient analyses were performed by Dairy One, Inc. (Ithaca, NY).

^2^Values are presented as an average analysis of nine bags of grain for entire experiment.

^3^Offspring were fed Creep Feed until day 120 of age.

^4^Offspring were fed Grower Feed from day 121 to 153 of age.

^5^Offspring were fed Complete Feed from day 154 to 282 of age. Values are presented as an average analysis of four deliveries.

### Sample Collection

Body weights and BCS of F0 ewes were recorded once per week during gestation until parturition. Lamb BW and jugular blood samples (10 to 20 mL) were collected weekly for the first 4 wk and then every 14 d until day 252 of age. Crown rump length (CRL), heart girth (HG), and hip-height measurements were taken at 0, 7, and 120 d of age. Lamb BCS were recorded at d 120 of age for all offspring and at 10 mo of age for ram offspring.

Blood samples were partitioned into heparin- (3 mL) and EDTA- (3 mL) coated tubes or tubes (10 mL) that did not contain an anticoagulant (Greiner Bio-one, Kremsmünster, Austria) for plasma and serum collection, respectively. Serum tubes were kept at room temperature for 4 to 6 h to allow blood to clot, then stored overnight at 4 °C and centrifuged the next morning. Plasma tubes were kept on ice until centrifugation, which was completed within 1 h of collection. Blood was centrifuged for 30 min at 3,000 × *g* (Eppendorf, Hamburg, Germany) at 4 °C, and serum and plasma were aliquoted and stored at −20 °C until analyzed.

Ram lambs were euthanized at day 282 ± 1.82 of age (Figure 1) by intravenous injection of Euthasol (Virbac, Fort Worth, TX) containing 390 mg/mL sodium pentobarbital and 50 mg/mL sodium phenytoin based on BW of the ram on the day of euthanasia (0.22 mL/kg BW). Immediately before euthanasia, CRL, HG, hip height, scrotal circumference, and BCS were measured, and a final blood sample (20 mL) was collected. After euthanasia, loin eye area (LEA; cm^2^) and backfat thickness were measured. Longissimus muscle (LM), semitendinosus (STN) and triceps brachii (TB) muscles, and heart, liver, pancreas, spleen, adrenal glands, kidneys, and testes were removed and weighed. The right hind leg of each ram was removed for length and bone density analyses. Density was determined using a dual-energy x-ray absorptiometry (DXA; Lunar Prodigy; GE Healthcare, Madison, WI) scanner which generated bone mineral density values (g/cm^2^) for each individual leg.

### Intravenous Glucose Tolerance Test

A fasting intravenous glucose tolerance test (IV-GTT) was performed at day 133 ± 0.25 of age. The necks of lambs were shaved and cleaned with chlorhexidine (Durvet, Beaver Dam, WI) followed by 70% ethanol (Fisher Bioreagents, Pittsburgh, PA). A cannula (18 g × 2.5 in; Exel International, Quebec, Canada) was inserted into a jugular vein of each lamb 1 h before GTT to allow lambs time to recover. A single bolus injection of glucose (0.25 g/kg BW of a 50% dextrose solution; VetOne, Boise, ID) was infused via the jugular cannula. Blood samples (3 mL) were collected via the cannula at −30, −15, 0, 2, 5, 10, 15, 30, 60, and 120 min relative to glucose infusion, placed into heparin tubes (Greiner Bio-one), and stored on ice. Blood was centrifuged (3,000 × *g* for 30 min at 4 °C), and plasma was stored at −20 °C for insulin and glucose analysis.

### Residual Feed Intake

At 120 ± 0.12 d of age, a radio frequency identification ear tag (Allflex, Rathway, NJ) was placed in the right ear of each lamb following manufacturer placement guidelines. At day 167 ± 1.42 of age, feed intake from each individual animal was measured for a 77-d feeding period to determine RFI (Koch et al., 1963; Arthur et al., 2001; Herd and Arthur, 2009; Cockrum et al., 2013) in which the animals had ad libitum access to a complete pelleted feed. Body weights on two consecutive days were measured at the beginning, mid-point, and end of the feeding trial. Average daily feed intake was calculated as total feed/days on feed. Feed conversion efficiency was calculated as total body weight gained/feed consumed during the feeding period. Predicted feed intake was calculated by regressing the actual (measured) feed intake on metabolic midweight [MMWT; (Mid-BW)^0.75^] and average daily gain [(final BW – starting BW)/days on feed]. Residual feed intake was calculated by subtracting the actual feed intake minus the predicted feed intake value that was calculated using the regression equation as previously described (Arthur et al., 2001). A negative RFI coefficient indicates that the animal consumed less than the predicted and is therefore more feed efficient.

### Circulating Factors Analyses

Circulating concentrations of total cholesterol (TC), triglyceride (TG), and leptin were determined using a commercially available cholesterol reagent set (Pointe Scientific, Canton, MI), L-Type TG M kit (Fujifilm Wako Diagnostics, Mountain View, CA), and multi-species radioimmunoassay (RIA; MilliporeSigma, Burlington, MA), respectively. The limit of detection for the TC and TG assays were 1.0 mg/dL and 1.1 mg/dL, respectively. The limit of detection for the leptin RIA was 0.801 ng/mL, and the intra- and inter-assay coefficients were 2.15% and 2.48%, respectively. These kits have been successfully optimized for use with ovine samples (Hoffman et al., 2014; Soranno et al., 2021), and manufacturer instructions were followed. Plasma and serum samples from day 0, 7, 14, 56, 210, and 252 of age were selected for TC, TG and leptin analyses to evaluate circulating concentrations at both pre-weaning (0, 7, 14, 56) and mature (210, 252) timepoints. Plasma samples from the IV-GTT were analyzed for insulin and glucose at all collected time points. Plasma insulin concentrations were determined by an ovine insulin enzyme-linked immunoassay (ELISA; Mercodia, Inc., Uppsala, Sweden) as previously described (Vaughan et al., 2016). The limit of detection for the insulin ELISA was 0.025 ng/mL, and the intra- and inter-assay coefficients were 6.57% and 4.95%, respectively. Cubic spline analysis was performed using an online data analysis tool (MyAssays Ltd.) for determination of insulin concentrations. Plasma glucose concentrations were determined using a colorimetric assay kit (Cayman Chemical, Ann Arbor, MI) as previously described (Hoffman et al., 2016). The limit of detection for the glucose colorimetric assay was 0.23 mg/dL, and the intra-assay coefficient was 6.54%. For glucose analysis, plasma was diluted 1:10 for −30, −15, 0, 5, 10, 15, 30, 60, 120 min samples and 1:15 for 2 min sample.

### Statistical Analysis

Data were analyzed using the R programming language in the R Studio (version 4.2.2; R Core Team, 2021) on “Spotted Wakerobin” release for Windows, using the packages car (Fox and Weisberg, 2020), emmeans (Lenth et al., 2022), ggpubr (Kassambara, 2020), lme4 (Bates et al., 2015), nlme (Pinheiro et al., 2022), rstatix (Kassambara, 2022), and tidyverse (Wickham et al., 2019). Body weight, body morphometric, and circulating factors data were analyzed using a two-way or three-way mixed effects analysis of variance (ANOVA) to account for repeated measures with animal (random), maternal treatment (fixed), sex (fixed), and time/day (continuous) included in the model, where appropriate. Sire and litter size were initially included as fixed effects in offspring BW analysis and had no effect (*P* ≥ 0.13) on offspring growth and therefore were removed from the model. Predicted feed intake was obtained through regression analysis of ADG and MMWT on actual daily feed intake. Residual value between actual and predicted intake was used as the RFI coefficient as previously described (Herd and Arthur, 2009; Cockrum et al., 2013). Baseline concentrations, area under the curve (AUC), first-phase response, and insulin to glucose ratio were determined as previously described (Hoffman et al., 2016). Organ weights are expressed as g/kg BW to account for differences in offspring BW. Bone lengths were determined using ImageJ (version 1.53) and analyzed as a one-way ANOVA with maternal treatment as the fixed effect. Where appropriate, post hoc pairwise comparisons were made using emmeans. Statistical significance was considered at *P* ≤ 0.05 and a tendency at *P* > 0.05 and < 0.10.

## Results

### F0 Ewes

**Figure 2.**
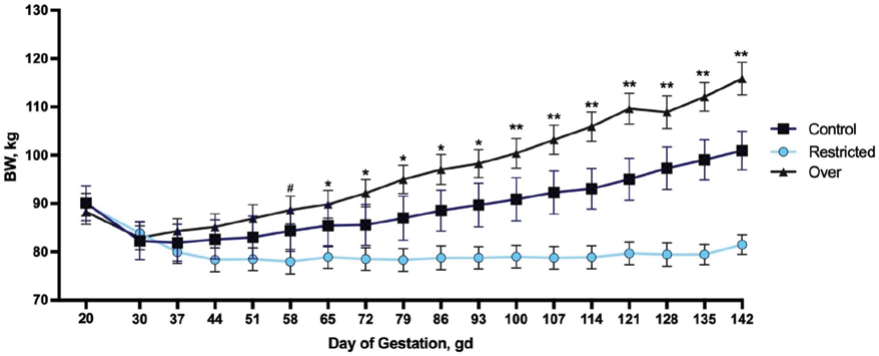
Body weight of F0 ewes. To evaluate the effects of restricted- and over-feeding during gestation on maternal BW, ewes were weighed at day 20 (before the start of dietary treatments), at day 30 (the day dietary treatment began) and weekly throughout gestation until parturition (day 147). ^#^*P* > 0.05 and < 0.10; ^*^P ≤ 0.05; ^**^P < 0.001.

Body weight (Figure 2) and BCS (data not shown) of F0 ewes did not differ before the dietary treatments began at day 20 of gestation (*P* = 0.89; *P* = 0.69) or from day 30 to day 51 of gestation (*P* = 0.16; *P* = 0.38). At day 58 of gestation, a tendency was observed for BW and BCS (*P* = 0.06; *P* = 0.08, respectively) where over-fed ewes were 13.6% heavier and had a 7.34% increase in BCS compared with restricted-fed ewes. Throughout gestation, restricted-fed ewes were 11.8% lighter than control-fed ewes and 20.3% lighter than over-fed ewes (*P* ≤ 0.0001; Figure 2). Similarly, throughout gestation, BCS was 15.5% and 14.3% less in restricted-fed ewes compared with control- and over-fed ewes, respectively (*P* ≤ 0.001).

### Offspring Growth and Body Morphometrics

At birth, RES offspring were 29.6% lighter than CON offspring (*P* ≤ 0.0001; Table 2), and OVER offspring did not differ from RES or CON (*P* ≥ 0.59). At day 7, RES and OVER offspring were 25.8% and 13.6% lighter than CON offspring, respectively (*P* ≤ 0.0001). From day 14 to 56, RES offspring tended to be 12.8% lighter than CON offspring (*P* ≤ 0.09) and OVER offspring did not differ from RES or CON (*P* ≥ 0.19). We did not detect a difference in offspring BW at day 70 (*P* = 0.14). From day 84 to 112, RES offspring were 11.5% lighter than CON offspring (*P* ≤ 0.02) and OVER offspring did not differ from RES or CON (*P* ≥ 0.52). From day 126 to 224, RES and OVER offspring were 10.8% and 7.8% lighter than CON offspring, respectively (*P* ≤ 0.006). From day 238 to 252, RES offspring tended to be 9.2% lighter than CON offspring (*P* ≤ 0.08) and OVER offspring did not differ from RES or CON (*P* ≥ 0.16). Additionally, ram lambs gained more BW (43.8 kg ± 0.8; *P ≤* 0.001) than ewe lambs (38.2 ± 0.7) from day 0 to 252 of age.

**Table 2.** Effects of maternal diet on offspring BW

	Treatment^1^				
Item^4^	CON	RES	OVER	SEM^2^	*P*-value^3^
BW, kg					
Day 0	5.30^a^	4.09^b^	4.73^ab^	0.28	<0.0001
Day 7	7.36^a^	5.85^b^	6.48^b^	0.33	<0.0001
Day 14	9.44^a^	7.86^b^	8.69^ab^	0.47	0.003
Day 21	11.54^a^	9.77^b^	10.67^ab^	0.60	0.016
Day 28	13.70^a^	11.80^b^	13.25^ab^	0.71	0.034
Day 42	19.15^x^	16.95^y^	18.70^xy^	0.97	0.069
Day 56	25.70^x^	23.00^y^	24.95^xy^	1.18	0.086
Day 70	31.00	28.25	29.60	1.29	0.138
Day 84	38.15^a^	33.75^b^	35.60^ab^	1.26	0.008
Day 98	43.50^a^	38.50^b^	40.35^ab^	1.57	0.017
Day 112	48.45^a^	42.90^b^	45.05^ab^	1.59	0.009
Day 126	52.05^a^	45.95^b^	47.75^b^	1.56	0.004
Day 140	54.85^a^	48.15^b^	50.20^b^	1.57	0.002
Day 154	57.90^a^	51.30^b^	52.90^b^	1.67	0.002
Day 168	61.05^a^	54.20^b^	56.25^b^	1.67	0.002
Day 182	64.90^a^	58.40^b^	59.45^b^	1.72	0.003
Day 196	69.10^a^	62.60^b^	63.85^b^	1.78	0.006
Day 210	72.60^a^	65.20^b^	67.65^b^	1.93	0.005
Day 224	77.20^a^	69.05^b^	71.60^b^	1.91	0.002
Day 238	80.80^a^	72.05^b^	76.55^ab^	2.16	0.003
Day 252	82.05^x^	75.80^y^	77.70^xy^	2.28	0.079

Means with different superscripts ^(a-b)^ within row represent differences among treatment (*P* ≤ 0.05).

Means with different superscripts ^(x-y)^ within row represent trend among treatment (*P* < 0.10).

^1^Dorset ewes pregnant with twins were fed 100%, 60%, or 140% of NRC requirements from d 30 of gestation until parturition. Offspring are referred to as CON, RES, and OVER, respectively.

^2^Largest SEM across treatments for each timepoint.

^3^ *P*-value for main effect of treatment at each timepoint.

^4^Offspring were weighed weekly for the first month and every 14 days until day 252 of age.

Body morphometric measurements were collected at day 0, 7, and 120. A three-way interaction was observed between treatment, sex, and day for CRL (*P* = 0.05). At day 0 and 7, CON (49.8 cm ± 1.3; 54.8 cm ± 1.4) and OVER (50.4 cm ± 1.32; 55.5 cm ± 1.4) rams had greater CRL than RES rams (*P* ≤ 0.04; 44.6 cm ± 0.8; 50.9 cm ± 1.2), and at day 120 CON rams (94.7 cm ± 1.4) and OVER rams (92.2 cm ± 1.3) had greater CRL than RES rams (*P* ≤ 0.05; 88.2 cm ± 1.1), and OVER rams did not differ CON rams (*P* = 0.40). At day 7, RES ewe lambs (50.6 cm ± 1.4) had smaller CRL compared with CON ewe lambs (*P* = 0.03; 56.0 cm ± 1.6) and OVER ewe lambs did not differ from RES or CON ewe lambs (*P* ≥ 0.29; 53.6 cm ± 1.32). Offspring from control-fed ewes (57.1 cm ± 0.6) had greater HG compared with both RES (54.0 cm ± 0.56) and OVER offspring (*P* = 0.01; 55.3 cm ± 0.6), and at day 120 rams (56.3 cm ± 0.5) had greater HG compared with ewes (*P* = 0.04; 54.6 cm ± 0.5). We did not detect an effect of maternal diet on body morphometric measurements (BW, BCS, CRL, HG, scrotal circumference, backfat thickness, and LEA) in rams at time of necropsy (*P* ≥ 0.13; Table 3). Organ weights were adjusted per kg BW to account for individual offspring BW. No effect of maternal diet was detected in offspring muscle weights (LM, STN, and TB; *P* ≥ 0.60). Similarly, heart, pancreas, spleen, adrenal gland, and kidney weights of offspring did not differ as a result of maternal diet (*P* ≥ 0.23; Table 3). Weight of liver from RES rams tended to be 14% heavier than livers from CON rams (*P* = 0.08). Testes weights from RES rams were 15.2% lighter than testes from CON rams (*P* = 0.05). Finally, ram tibia and femur length were 4.77% and 6.58% shorter, respectively, in RES rams compared with CON rams (*P* ≤ 0.05; Table 4). Tibia and femur length did not differ for OVER rams (*P* ≥ 0.17). Bone mineral density tended to be decreased in RES rams compared with CON rams (*P* = 0.06).

**Table 3.** Impact of poor maternal nutrition on ram offspring body morphometrics and organ weights at day 282 of age

	Treatment^1^				
Item^4^	CON	RES	OVER	SEM^2^	*P*-Value^3^
Body Morphometrics					
BW, kg	93.20	89.91	90.00	1.28	0.53
BCS	3.08	3.03	2.92	0.04	0.28
CRL, cm	117.90	114.33	117.63	0.82	0.13
HG, cm	114.64	110.94	112.10	0.79	0.17
Scrotal circumference, cm	35.05	34.00	34.17	0.37	0.52
Backfat, cm	0.18	0.19	0.20	0.00	0.66
LEA, cm^2^	117.90	109.11	120.29	2.90	0.24
Organ Weights^5^					
LM, g/kg BW	11.50	11.90	11.92	0.26	0.76
STN, g/kg BW	2.97	3.01	2.85	0.06	0.60
TB, g/kg BW	1.00	1.00	1.03	0.02	0.87
Heart, g/kg BW	4.50	4.28	4.50	0.08	0.43
Liver, g/kg BW	14.68^x^	17.08^y^	16.59^xy^	0.45	0.08
Pancreas, g/kg BW	0.52	0.51	0.55	0.02	0.72
Spleen, g/kg BW	4.50	4.20	4.32	0.18	0.80
Adrenal Gland, g/kg BW	0.02	0.02	0.03	0.00	0.23
Kidney, g/kg BW	1.72	1.81	1.66	0.05	0.52
Testes, g/kg BW	3.29^a^	2.79^b^	3.16^ab^	0.09	0.05

Means with different superscripts ^(a-b)^ within row represent differences among treatment (*P* ≤ 0.05).

Means with different superscripts ^(x-y)^ within row represent trend among treatment (*P* < 0.10).

^1^Dorset ewes pregnant with twins were fed 100%, 60%, or 140% of NRC requirements from day 30 of gestation until parturition. Offspring are referred to as CON, RES, and OVER, respectively. Male offspring were necropsied at day 282 of age for tissue collection.

^2^Largest SEM across treatments for each variable.

^3^ *P*-value for main effect of treatment.

^4^CRL, crown rump length; HG, heart girth; LEA, loin eye area; LM, longissimus muscle; STN, semitendinosus; TB, triceps brachii.

^5^Organ weights are expressed as g/kg BW to account for differences in BW between treatment groups.

**Table 4.** Right leg bone mineral density and length in male offspring at day 282

	Treatment^1^				
Item^4^	CON	RES	OVER	SEM^2^	*P*-Value^3^
Bone Mineral Density, g/cm^2^	1.12^x^	1.06^y^	1.11^xy^	0.02	0.06
Tibia Length, cm	87.87^a^	83.87^b^	86.17^ab^	1.48	0.05
Femur Length, cm	75.0^a^	70.38^b^	72.43^ab^	1.43	0.02

Means with different superscripts ^(a-b)^ within row represent differences among treatment (*P* ≤ 0.05).

Means with different superscripts ^(x-y)^ within row represent trend among treatment (*P* < 0.10).

^1^Dorset ewes pregnant with twins were fed 100%, 60% or 140% of NRC requirements from d 30 of gestation until parturition. Offspring are referred to as CON, RES, and OVER, respectively. Male offspring were necropsied at day 282 of age and the right leg was removed for density and length analysis.

^2^Largest SEM across treatments for each variable.

^3^ *P*-value for main effect of treatment.

^4^Bone mineral density values were obtained from DXA scanner (Lunar Prodigy; GE Healthcare, Madison, WI). Tibia and femur length were obtained from DXA scans using ImageJ.

### Residual Feed Intake Trial

Residual feed intake was determined in a 77-day feeding experiment for all lambs beginning on day 168 of age (Table 5). At the beginning of RFI, RES and OVER offspring were 11.6% and 10.0% lighter compared with CON offspring, respectively (*P* ≤ 0.013). The initial BW of the ewe lambs was also 15.7% less than the ram lambs (*P* ≤ 0.0001). Following the 77-d feeding trial, OVER ewes weighed 11.2% less than CON ewes (*P* ≤ 0.012), and RES rams weighed 12.3% and 8.8% less than CON and OVER rams, respectively (*P* ≤ 0.014). The MMWT of RES and OVER offspring was 7.7% and 6.5% less compared with CON offspring, respectively (*P* ≤ 0.012). Ewe offspring MMWT was 10.1% less than ram offspring MMWT (*P* ≤ 0.001). Although average daily gain was not impacted by maternal diet or sex of the offspring (*P* ≥ 0.28), RES and OVER ewe lambs tended to consume 9.3% and 11.2% less feed on average compared with CON ewes (*P* = 0.07). Despite the differences observed in average daily intake, there were no differences in the RFI coefficient detected in the offspring as a result of maternal diet (*P* = 0.57). Ram lambs had a lower RFI coefficient (−0.07 ± 0.03) than ewe lambs (0.11 ± 0.03; *P* ≤ 0.0007), indicating that ram lambs consumed less feed than expected and had greater feed efficiency.

**Table 5.** Effects of poor maternal nutrition on ewe and ram offspring residual feed intake

	Treatment^1^							*P*-Value		
	CON		RES		OVER					
Item^3^	Ewe	Ram	Ewe	Ram	Ewe	Ram	SEM^2^	Trt	Sex	Trt*Sex
Initial BW^4^, kg	55.0	65.23	49.82	56.36	48.13	60.01	2.91	0.001	<0.0001	0.35
Final BW^5^, kg	76.83^bc^	86.79^d^	70.61^ab^	76.09^bc^	68.24^a^	83.42^cd^	2.76	<0.0001	<0.0001	0.04
MMWT^6^, kg	23.44	25.91	21.87	23.75	21.6	24.63	0.74	0.001	<0.0001	0.50
ADG, kg/d	0.28	0.28	0.27	0.26	0.26	0.30	0.02	0.28	0.49	0.14
Average Daily Intake^7^, kg/d	3.21^x^	3.07^xy^	2.94^xy^	2.86^y^	2.85^y^	3.08^xy^	0.11	0.02	0.88	0.07
RFI coefficient^8^^,^^9^, kg/d	0.22	-0.10	0.08	-0.10	0.04	-0.04	0.08	0.57	0.001	0.20

Means with different superscripts ^(a-c)^ within row represent differences among treatment (*P* ≤ 0.05)

Means with different superscripts ^(x-y)^ within row represent trend among treatment (*P* < 0.10)

^1^Dorset ewes pregnant with twins were fed 100%, 60%, or 140% of NRC requirements from day 30 of gestation until parturition. Offspring are referred to as CON, RES, and OVER, respectively. Offspring were transitioned to a complete pelleted feed at day 153 of age. At day 168 of age, offspring were allowed ad libitum feed intake for a 77-day residual feed intake trial.

^2^Largest SEM across treatments for each variable.

^3^MMWT, metabolic mid-weight; RFI, residual feed intake.

^4^Average of two consecutive body weights at the beginning of the trial.

^5^Average of two consecutive body weights at the end of the trial.

^6^Mid-point BW^0.75^

^7^Daily intake values averaged across the 77-day feeding trial.

^8^Coeffieicent [Average daily intake – Predicted daily intake] where predicted daily intake is obtained by the regression of average daily intake on MMWT and ADG (Herd and Arthur, 2009).

^9^A negative value indicates a more efficient animal.

### Circulating Metabolic Factors

Circulating concentrations of leptin (Pre-weaning; Table 6), TC, and TG were measured at day 0, 7, 14, and 56 of age (Pre-weaning; Table 7). Pre-weaning offspring from over-fed ewes tended to have 70.2% greater circulating leptin concentrations compared with CON offspring (*P* = 0.07; Table 6), and RES offspring did not differ from CON or OVER (*P* ≥ 0.44). Offspring sex did not affect pre-weaning offspring leptin concentrations (*P* ≥ 0.22). Maternal diet did not affect pre-weaning circulating TC or TG (*P* ≥ 0.35; Table 7). However, offspring sex tended to have an effect on circulating concentrations of TC and TG (*P* ≤ 0.07). A tendency for a sex by day interaction was observed where ewe lambs tended to have 12.5% and 9.0% less circulating TC at day 7 and day 14 compared with ram lambs, respectively (*P* ≤ 0.07). Ewe lambs also tended to have 11.5% less circulating TG compared with ram lambs when averaged across all pre-weaning time points (*P* = 0.07).

**Table 6.** Concentrations of leptin in CON, RES, and OVER ewe and ram lamb serum at day 0, 7, 14, 56, 210, and 252 of age

	Treatment^1^							*P*-Value						
	CON		RES		OVER									
Item	Ewe	Ram	Ewe	Ram	Ewe	Ram	SEM^2^	Trt	Sex	Day	Trt*Sex	Trt*Day	Sex*Day	Trt*Sex*Day
*Pre-Weaning* ^3^														
Leptin, ng/dL								0.07	0.67	0.002	0.36	0.81	0.22	0.72
Day 0	2.11^a^	1.30^a^	1.80^ab^	3.48^ab^	2.82^b^	3.25^b^	1.20							
Day 7	1.35^a^	1.07^a^	1.12^ab^	2.48^ab^	1.93^b^	2.20^b^	0.79							
Day 14	1.39^a^	0.89^a^	1.19^ab^	1.47^ab^	2.30^b^	2.07^b^	0.59							
Day 56	1.87^a^	1.31^a^	2.21^ab^	1.77^ab^	2.57^b^	2.15^b^	0.66							
*Maturity* ^4^														
Leptin, ng/dL								0.12	<0.0001	<0.0001	0.68	0.63	0.85	0.06
Day 210	15.68^bc^	7.93^ab^	14.34^bcd^	5.59^a^	14.56^bcd^	6.10^a^	1.99							
Day 252	21.77^d^	9.01^abc^	15.98^bcd^	8.35^ab^	15.97^cd^	8.49^ab^	1.92							

Means with different superscripts ^(a-c)^ within row and column represent trend among treatment (*P* < 0.10).

^1^Dorset ewes pregnant with twins were fed 100%, 60%, or 140% of NRC requirements from day 30 of gestation until parturition. Offspring are referred to as CON, RES, and OVER, respectively. Blood samples were collected from offspring weekly for the first month of life and every 14 d after until day 252 of age. A subset of timepoints were analyzed for each animal.

^2^Largest SEM across treatments for each variable.

^3^Day 0, 7, 14, and 56

^4^Day 210 and 252

**Table 7.** Concentrations of total cholesterol and triglyceride in CON, RES, and OVER ewe and ram lamb plasma at day 0, 7, 14, 56, 210, 252 of age

	Treatment^1^							*P*-Value		
	Control		Restricted		Over					
Item	Ewe	Ram	Ewe	Ram	Ewe	Ram	SEM^2^	Trt	Sex	Day
*Pre-Weaning* ^3^										
Total Cholesterol, mg/dL								0.43	0.13	<0.0001
Day 0	43.10	40.01	36.19	45.95	44.51	44.98	4.37			
Day 7	84.37	94.48	68.20	88.44	78.81	82.21	8.49			
Day 14	117.57	128.84	113.72	114.79	100.07	121.01	15.53			
Day 56	53.72	50.86	67.27	53.73	63.86	59.10	7.96			
Triglyceride, mg/dL								0.35	0.07	<0.0001
Day 0	40.71	25.64	23.59	34.86	38.83	35.60	8.42			
Day 7	29.20	29.62	33.11	32.81	26.35	33.68	3.92			
Day 14	32.87	46.11	34.08	50.33	46.80	47.19	7.45			
Day 56	21.84	32.15	20.74	27.45	28.12	30.69	4.39			
*Maturity* ^4^										
Total Cholesterol, mg/dL								0.64	0.11	0.002
Day 210	62.31	55.49	61.57	64.47	65.47	54.58	6.43			
Day 252	71.94	69.39	72.79	66.05	82.05	69.53	8.79			
Triglyceride, mg/dL								0.71	0.04	0.39
Day 210	15.37	15.72	12.31	17.40	16.10	15.79	1.60			
Day 252	14.84	19.25	16.71	17.70	14.17	16.09	2.51			

^1^Dorset ewes pregnant with twins were fed 100%, 60%, or 140% of NRC requirements from d 30 of gestation until parturition. Offspring are referred to as CON, RES, and OVER, respectively. Blood samples were collected from offspring weekly for the first month of life and every 14 d after until day 252 of age. A subset of timepoints were analyzed for each animal.

^2^Largest SEM across treatments for each variable.

^3^Days 0, 7, 14, and 56.

^4^Days 210 and 252.

Circulating concentrations of leptin (Maturity; Table 6), TC, and TG were also evaluated in offspring at day 210 and 252 of age (Maturity; Table 7). A tendency for a three-way interaction between treatment, sex, and day was observed for leptin concentrations where CON ewes tended to have 2.4-fold greater leptin concentration compared with CON rams, 1.4-fold greater compared with RES ewes, 2.5-fold greater compared with RES, 1.37-fold greater compared with OVER ewes, and 2.5-fold greater compared with OVER rams at day 252 (*P* = 0.06; Table 6). No effect of maternal diet or sex were observed for TC concentrations in mature animals (*P* ≥ 0.11; Table 7). Similar to pre-weaning TG concentrations, mature ewes had 12.4% less circulating TG concentrations compared with mature rams (*P* = 0.04), though no effect of maternal diet on offspring TG concentrations were detected (*P* = 0.71).

### Intravenous Glucose Tolerance Test

Plasma glucose and insulin concentrations were measured from an IV-GTT performed on lambs at day 133 of age (Table 8). Glucose and insulin concentrations were increased in all animals within 2 min of glucose infusion and returned to pre-infusion concentrations by 120 min post-infusion (Table 8). A treatment by time interaction was observed for glucose response to glucose challenge (*P* = 0.002). At 2 min following glucose infusion, glucose concentrations were greater in OVER offspring (*P* < 0.05; 209.9 mg/dL ± 6.3) compared with CON offspring (194.2 mg/dL ± 7.6). Additionally, a tendency for a three-way interaction between treatment, sex, and time was observed for insulin response to glucose challenge (*P* = 0.08). At both 5 and 10 min relative to glucose infusion, insulin concentrations in ram offspring from restricted-fed ewes tended to be greater (*P* ≤ 0.07; 1.22 mg/dL ± 0.08; 1.52 mg/dL ± 0.08) compared with ewe offspring from restricted-fed ewes (0.82 mg/dL ± 0.10; 1.12 mg/dL ± 0.10) and ewe offspring from over-fed ewes (0.88 mg/dL ± 0.09; 1.18 mg/dL ± 0.09). At 15 min relative to glucose infusion, insulin concentrations in ram offspring from restricted-fed ewes tended to be greater (*P* ≤ 0.07; 1.27 mg/dL ± 0.08) compared with ewe offspring from restricted-fed ewes (0.89 mg/dL ± 0.10). Despite these effects of maternal diet at individual time points during the glucose tolerance challenge, we did not detect a difference among treatment groups (*P* ≥ 0.15) for glucose or insulin concentrations averaged over the challenge, baseline glucose or insulin concentrations, peak glucose or insulin concentrations, AUC of glucose or insulin, first-phase insulin response, or insulin to glucose ratios. Offspring sex tended to impact peak insulin concentrations, first phase insulin response, and insulin to glucose ratio. Specifically, rams tended to have greater peak insulin concentration (*P* = 0.07; 1.35 mg/dL ± 0.08) compared with ewes (1.18 mg/dL ± 0.08). Rams also had greater first phase insulin response (*P* = 0.04; 2.90 ng/dL ± 0.16) compared with ewes (2.47 ng/dL ± 0.18). Finally, rams tended to have greater insulin to glucose ratio (*P* = 0.09; 0.0066 ± 0.0003) compared with ewes (0.0057 ± 0.0004).

**Table 8.** Plasma glucose and insulin concentrations from the intravenous glucose tolerance test performed on CON, RES, and OVER ewe and ram lambs at day 133 of age

		Treatment^1^							*P*-Value		
Item		CON		RES		OVER					
Glucose		Ewe	Ram	Ewe	Ram	Ewe	Ram	SEM^2^	Trt	Sex	Trt*Sex
	Average of all time points, mg/dL	122.00	114.00	122.00	112.00	116.00	123.00	5.93	0.80	0.40	0.17
	Baseline^3^, mg/dL	73.60	69.00	69.20	67.30	68.70	67.60	3.43	0.54	0.33	0.84
	Peak concentration, mg/dL	193.00	195.00	200.00	190.00	204.00	216.00	11.73	0.15	0.91	0.66
	AUC^4^, AU	10,957.60	10,452.71	10,957.32	10,168.70	10,341.69	11,033.52	678.69	0.91	0.68	0.29
Insulin											
	Average of all time points, ng/dL	0.64	0.65	0.65	0.85	0.71	0.74	0.09	0.33	0.14	0.35
	Baseline^3^, ng/dL	0.24	0.28	0.29	0.30	0.32	0.31	0.48	0.43	0.72	0.18
	Peak concentration, ng/dL	1.24	1.17	1.12	1.56	1.19	1.32	0.16	0.48	0.08	0.20
	AUC^4^, AU	71.49	83.83	79.07	97.13	88.63	86.12	15.16	0.57	0.31	0.59
	First-phase response^5^, ng/mL	2.59	2.47	2.38	3.46	2.44	2.77	0.34	0.23	0.04	0.13
	Insulin, ng/mL; glucose ratio, mg/dL	0.005	0.006	0.005	0.008	0.006	0.006	0.001	0.39	0.09	0.23

^1^Dorset ewes pregnant with twins were fed 100%, 60%, or 140% of NRC requirements from day 30 of gestation until parturition. Offspring are referred to as CON, RES, and OVER, respectively. Offspring were subjected to IVGTT at day 133 of age.

^2^Largest SEM across treatments for each variable.

^3^Determined from plasma samples collected at −30, −15, and 0 min relative to glucose infusion.

^4^AUC, area under the curve; AU, arbitrary units.

^5^First-phase response was calculated as the sum of 2, 5, and 10 min insulin concentrations post-glucose infusion subtracted by the mean baseline insulin concentration.

## Discussion

As expected, ewes fed 100% of their nutrient requirements had increased BW throughout gestation with a greater increase during late gestation, whereas ewes fed 60% of their nutrient requirements did not gain throughout gestation and had reduced BCS in late gestation, likely due to utilization of fat reserves. Ewes fed 140% of their nutrient requirements had greater BW gain than control-fed and restricted-fed ewes throughout gestation and increased BCS compared with restricted-fed ewes, demonstrating storage of excess nutrients. All ewes regardless of diet experienced BW loss during the 10 d transition period which is consistent with our previous studies (Reed et al., 2014; Hoffman et al., 2016; Pillai et al., 2017; Jones et al., 2018). The differences in maternal BW throughout gestation are also consistent with our previous reports using this model of poor maternal nutrition demonstrating the effectiveness of the diets.

Maternal diet during gestation influences offspring growth and fetal development as we have previously reported (Reed et al., 2014; Hoffman et al., 2016; Pillai et al., 2017); however these reports were limited to only fetal or early postnatal timepoints, and evaluations of offspring through maturity in livestock are limited. Offspring from over-fed ewes were of similar BW from 2 to 19 mo of age, but during an ad libitum feeding challenge, offspring from over-fed ewes gained more than offspring from control-fed ewes (Long et al., 2010). Male offspring from restricted-fed ewes were reported to be heavier at maturity compared with control-fed ewes, indicating compensatory gain in these offspring (Zhu et al., 2006; Ford et al., 2007). Contrary to these reports, the present study found that offspring from both restricted- and over-fed dams weighed less compared with offspring from control-fed dams from 4.5 to 8 mo of age. During the ad libitum feeding period from 6 to 8.5 mo of age, RES and OVER offspring consumed less feed than CON offspring. Therefore, some differences in BW may be attributed to differences in feed intake. However, the present study only monitored individual feed intake during the RFI period, thus feed intake during other time points (early postnatal and maturity) are unknown as the animals were group housed. Although these offspring consumed less feed, their RFI was not influenced by maternal diet, suggesting that another factor may be influencing the growth of these offspring. Evaluating RFI at a time corresponding to the BW differences in the offspring or during an earlier period of rapid growth may be important times to also evaluate feed efficiency.

To further evaluate the effects of maternal diet on offspring growth, we investigated offspring morphometrics and organ weights; however, due to the design of this study, we were only able to evaluate organ and bone development in the male offspring. Decreased testicular size is associated with poor fertility as the smaller size can change the rate of sperm production (Martin et al., 1994). Decreased testes weight in offspring, as a result of maternal energy restriction during gestation has been reported in pig (Lin et al., 2019) and sheep (Hoffman et al., 2018) models. Testes weight in these studies corresponded with decreased Sertoli and Leydig cell number which are critical for spermatogenesis (Sharpe et al., 1981). However, males in these studies were 2 mo of age or younger and therefore not at sexual maturity. In the present study, we evaluated rams at 10 mo of age and found that rams from restricted-fed ewes had decreased testes weight compared with rams from control-fed ewes. Although, scrotal circumference is an accepted tool for breeding soundness exams (Söderquist and Hultén, 2006), in the current experiment the decrease in testes weight was observed independent of scrotal circumference. The rams in the current study all had scrotal circumferences ≥ 34 cm which is an acceptable size for use as a breeding ram (Maquivar et al., 2021). Further investigation is warranted to determine if decreased testes weight at maturity corresponds with reduced fertility, due to sperm physiology and(or) morphology as this could have negative consequences on the livestock industry, specifically in breeding animals.

Decreased BMD at an early age can increase the risk of development of bone related diseases such as osteoporosis or osteoporotic fracture (Sayer and Cooper, 2005). Bone fracture creates both economical and welfare concerns in livestock industries (Gibson et al., 2020). Neural and bone tissue grow with highest priority during fetal development (Steele and Pursel, 1990); when maternal nutritional plane shifts to a restricted environment, nutrients may be partitioned away from bone toward neural development, resulting in under-developed bones. Maternal protein restriction in rats results in decreased bone mineral content (Mehta et al., 2002), altered length in male and female offspring, and decreased femoral head and tibia midshaft strength in female offspring (Lanham et al., 2008). Neither report resulted in altered BMD and investigations on the effect of poor maternal diet using livestock models are limited. Previous work from our lab did not detect an effect of restricted- or over-feeding during gestation on offspring BMD or femur and tibia length at day 1 or 3 mo of age (Pillai et al., 2016). However, in the present study, we see an effect of maternal diet on ram BMD, tibia length, and femur length at 10 mo of age, suggesting that there is likely programming during gestation that is impacting the development of this key structural tissue that may have negative impacts as the lambs age, and as they approach maturity.

Previous work from our lab and others report decreased fetal liver weight in offspring from restricted-fed dams (Xue et al., 2019; Smith et al., 2021; Muroya et al., 2022). Interestingly, we report that rams from restricted-fed ewes had increased liver weight compared with ram livers from control-fed ewes. There are limited reports on the effects of maternal diet on offspring liver size postnatally, therefore this area warrants further investigation to determine if differences in postnatal liver size correlate to metabolic status. Altered metabolism in offspring as a result of maternal diet has been demonstrated across species (Barker, 1997; Benyshek et al., 2006; Muhlhausler et al., 2007; Altmann et al., 2012; Smith et al., 2021). However, there is limited research on metabolic variables in offspring at maturity. The mature timepoint is critical in livestock models due to the age/weight that animals are marketed.

We investigated circulating leptin at both pre-weaning and mature timepoints due to the role of leptin in appetite regulation, relationship with adiposity, and previous research that demonstrates its sensitivity to poor maternal diet (Long et al., 2010, 2011; Hoffman et al., 2014; Shasa et al., 2015; Dornbush and Aeddula, 2023). As expected, offspring from over-fed ewes tended to have increased pre-weaning leptin concentrations compared with CON offspring. These findings are supported by our previous reports of increased leptin in offspring from over-fed ewes (Hoffman et al., 2014, 2016). However, at maturity we observed a tendency that RES and OVER rams tended to have decreased leptin relative to CON ewes. Due to the relationship between appetite and adiposity, we suspect that this may be explained by the differences in body weight and feed intake that we observed in these offspring. Circulating TC and TG are essential for cellular structure and energy metabolism (Cox and Garcia-Palmieri, 1990). However, in excess these circulating factors can have negative consequences on health. We have previously reported that TC and TG concentrations were not influenced by maternal diet at day 1 or 3 mo of age (Hoffman et al., 2016), which are similar to our findings in the present study. Rams had greater circulating TG at both pre-weaning and mature timepoints and greater TC at day 7 and 14 compared with ewe lambs. Sex-specific increases in plasma lipids are likely due to differences in BW between rams and ewes. Poor maternal diet can negatively impact offspring glucose tolerance by increasing insulin:glucose and AUC (Ford et al., 2007; Long et al., 2015; Hoffman et al., 2016). Contrary to our hypothesis, maternal diet did not impact offspring insulin:glucose, glucose AUC, insulin AUC, or first-phase insulin response. However, at 5, and 10 min post glucose infusion, RES rams had increased insulin concentrations compared with RES and OVER ewes, demonstrating that RES rams required greater concentrations of insulin in response to the glucose bolus than ewes. The present study reports clear effects of sex, but minimal changes in select circulating factors and offspring glucose tolerance as a result of maternal diet. These findings are surprising given the effect of maternal diet on offspring BW.

In conclusion, we determined that maternal restricted- and over-feeding during gestation alter growth of male and female offspring from birth through maturity, independent of differences in feed efficiency (RFI), glucose tolerance, and select circulating metabolic factors. Maternal diet also impacts mature ram BMD, bone length, testes weight, and liver weight. Future investigation is warranted to investigate mechanisms of impaired growth, and if the effects of maternal diet on offspring growth and metabolism persist across subsequent generations.
