# Correction to: Restricted- and over-feeding during gestation decreases growth of offspring throughout maturity

**DOI:** 10.1093/tas/txaf135

**Published:** 2025-10-24

This is a correction to: Nicole M Tillquist, Sarah A Reed, Mia Y Kawaida, Amanda S Reiter, Brandon I Smith, Hyung Jang, Ji-Young Lee, Elaine C Lee, Steven A Zinn, Kristen E Govoni, Restricted- and over-feeding during gestation decreases growth of offspring throughout maturity, *Translational Animal Science*, Volume 7, Issue 1, 2023, txad061, https://doi.org/10.1093/tas/txad061

In the originally published online version of this manuscript, data Table 3 was errored being reported to be in cm instead of inches, while LEA is off by a factor of 10. Table 3 should read:

There are no changes to the findings of the paper.

The emendation has been outlined only in this correction notice to preserve the version of record.

**Table 3.** Impact of poor maternal nutrition on ram offspring body morphometrics and organ weights at d 282 of age

Item^4^	Treatment^1^				
	CON	RES	OVER	SEM^2^	*P*-Value^3^
Body Morphometrics					
BW, kg	93.20	89.91	90.00	1.28	0.53
BCS	3.08	3.03	2.92	0.04	0.28
CRL, cm	117.90	114.33	117.63	0.82	0.13
HG, cm	114.64	110.94	112.10	0.79	0.17
Scrotal circumference, cm	35.05	34.00	34.17	0.37	0.52
Backfat, cm	0.45	0.48	0.49	0.03	0.66
LEA, cm2	38.00	35.20	38.80	1.75	0.24
Organ Weights^5^					
LM, g/kg BW	11.50	11.90	11.92	0.26	0.76
STN, g/kg BW	2.97	3.01	2.85	0.06	0.60
TB, g/kg BW	1.00	1.00	1.03	0.02	0.87
Heart, g/kg BW	4.50	4.28	4.50	0.08	0.43
Liver, g/kg BW	14.68^x^	17.08^y^	16.59^xy^	0.45	0.08
Pancreas, g/kg BW	0.52	0.51	0.55	0.02	0.72
Spleen, g/kg BW	4.50	4.20	4.32	0.18	0.80
Adrenal Gland, g/kg BW	0.02	0.02	0.03	0.00	0.23
Kidney, g/kg BW	1.72	1.81	1.66	0.05	0.52
Testes, g/kg BW	3.29^a^	2.79^b^	3.16^ab^	0.09	0.05

Means with different superscripts ^(a-b)^ within row represent differences among treatment (*P* ≤ 0.05).

Means with different superscripts ^(x-y)^ within row represent trend among treatment (*P* < 0.10).

1 Dorset ewes pregnant with twins were fed 100%, 60% or 140% of NRC requirements from d 30 of gestation until parturition. Offspring are referred to as CON, RES, and OVER, respectively. Male offspring were necropsied at d 282 of age for tissue collection.

2 Largest SEM across treatments for each variable.

3 *P*-value for main effect of treatment.

4 CRL, crown rump length; HG, heart girth; LEA, loin eye area; LM, longissimus muscle; STN, semitendinosus; TB, triceps brachii.

5 Organ weights are expressed as g/kg BW to account for differences in BW between treatment groups.

